# PprM, a Cold Shock Domain-Containing Protein from *Deinococcus radiodurans*, Confers Oxidative Stress Tolerance to *Escherichia coli*

**DOI:** 10.3389/fmicb.2016.02124

**Published:** 2017-01-10

**Authors:** Sun-Ha Park, Harinder Singh, Deepti Appukuttan, Sunwook Jeong, Yong Jun Choi, Jong-Hyun Jung, Issay Narumi, Sangyong Lim

**Affiliations:** ^1^Research Division for Biotechnology, Korea Atomic Energy Research InstituteJeongeup, South Korea; ^2^Radiation Microbiology Laboratory, Department of Life Sciences, Faculty of Life Sciences, Toyo UniversityGunma, Japan

**Keywords:** *Deinococcus radiodurans*, PprM, *Escherichia coli*, oxidative stress tolerance, MntH (proton-dependent Mn transporter), *ycgA*-*ymgABC* operon

## Abstract

*Escherichia coli* is a representative microorganism that is frequently used for industrial biotechnology; thus its cellular robustness should be enhanced for the widespread application of *E. coli* in biotechnology. Stress response genes from the extremely radioresistant bacterium *Deinococcus radiodurans* have been used to enhance the stress tolerance of *E. coli*. In the present study, we introduced the cold shock domain-containing protein PprM from *D. radiodurans* into *E. coli* and observed that the tolerance to hydrogen peroxide (H_2_O_2_) was significantly increased in recombinant strains (Ec-PprM). The overexpression of PprM in *E. coli* elevated the expression of some OxyR-dependent genes, which play important roles in oxidative stress tolerance. Particularly, *mntH* (manganese transporter) was activated by 9-fold in Ec-PprM, even in the absence of H_2_O_2_ stress, which induced a more than 2-fold increase in the Mn/Fe ratio compared with wild type. The reduced production of highly reactive hydroxyl radicals (·OH) and low protein carbonylation levels (a marker of oxidative damage) in Ec-PprM indicate that the increase in the Mn/Fe ratio contributes to the protection of cells from H_2_O_2_ stress. PprM also conferred H_2_O_2_ tolerance to *E. coli* in the absence of OxyR. We confirmed that the H_2_O_2_ tolerance of *oxyR* mutants reflected the activation of the *ycgZ*-*ymgABC* operon, whose expression is activated by H_2_O_2_ in an OxyR-independent manner. Thus, the results of the present study showed that PprM could be exploited to improve the robustness of *E. coli*.

## Introduction

*Escherichia coli* (*E. coli*) is one of the most frequently used bacterial hosts for the industrial production of recombinant proteins (Gopal and Kumar, 2013), biofuels (Clomburg and Gonzalez, 2010), and pharmaceuticals (Ferrer-Miralles et al., 2009). Rapid growth rates, high cell-density fermentation, and the availability of various genetic tools are crucial factors that facilitate the industrial use of *E. coli* (Makino et al., 2011). However, the production and accumulation of recombinant proteins, fuels, and chemicals can induce a variety of stresses in *E. coli*, such as an unsuitable pH, temperature, osmotic pressure, and oxidative stress, all of which reduce the production from this bacteria (Han et al., 2001; Hoffmann and Rinas, 2004; Rutherford et al., 2010). Oxidative stress induced through reactive oxygen species (ROS), such as superoxide radical (${\text{O}}_{2}^{-}$ ·), hydroxyl radical (·OH), and hydrogen peroxide (H_2_O_2_), is common because ROS inevitably results from aerobic growth in a fermenter (Li et al., 2009). ROS has harmful effects on cells, including DNA mutations, metabolic pathway disruption, and growth inhibition (Imlay, 2013). Therefore, oxidative stress tolerance is a key characteristic for industrial host strains, and various methods have been explored to enhance the tolerance to oxidative stress (Basak and Jiang, 2012; Lee et al., 2014; Zhao et al., 2014).

*Deinococcus radiodurans* (*D. radiodurans*) is well known for its ability to survive multiple extreme stresses, including gamma radiation (γ-radiation), UV light, ROS, and other DNA-damaging agents (Slade and Radman, 2011). Various enzymatic and non-enzymatic anti-oxidative systems, redundancy in DNA repair genes with a unique DNA repair machinery called extended synthesis dependent strand annealing (ESDSA), and some physical characteristics of nucleoids, which are advantageous to DNA repair, contribute to the extreme resistance of *D. radiodurans* (Ishino and Narumi, 2015; Munteanu et al., 2015). However, the precise mechanisms governing the multiple resistance characteristics of this organism remain unclear. Stress responsive genes from *D. radiodurans* have been used to enhance the stress tolerance of *E. coli*. Indeed, the heterologous expression of a pyrrolquinoline-quinone (PQQ) synthase, a manganese (Mn) transporter protein (MntH), a small heat shock protein (Hsp20), and a response regulator (DR1558) in *E. coli* improved oxidative stress tolerance (Khairnar et al., 2003; Haiyan and Baoming, 2010; Singh et al., 2014; Appukuttan et al., 2016).

*Deinococcus*-specific *ppr* (pleiotropic protein promoting DNA repair) genes are essential for the extreme resistance of this organism (Hua et al., 2003; Narumi et al., 2004). A global regulator, PprI (also named IrrE), serves as a general switch for downstream DNA repair and protection pathways (Lu et al., 2009). The introduction of native and engineered PprI has been effective in not only enhancing the tolerance of *E. coli* against abiotic stresses, including oxidative stress (Gao et al., 2003), but also in improving ethanol production in ethanologenic *E. coli* (Pan et al., 2009; Ma et al., 2011). PprA, which plays a role in DNA damage resistance and the genome maintenance of *D. radiodurans* (Devigne et al., 2013; Selvam et al., 2013; Kota et al., 2014), also enhanced tolerance against oxidative stress when expressed in *E. coli* (Kota and Misra, 2006). PprM, a cold shock protein (CSP) homolog in *D. radiodurans*, functions as a novel modulator of PprA in PprI-dependent signal transduction pathways, and the deletion of *pprM* reduces resistances to γ-radiation (Ohba et al., 2009) and H_2_O_2_ stress (Jeong et al., 2016b). Taken together, these observations prompted us to investigate the effect of PprM on oxidative stress tolerance in *E. coli*.

In the present study, we observed that *E. coli* cells expressing PprM exhibited improved tolerance to hydrogen peroxide (H_2_O_2_) through an increased intracellular Mn/Fe ratio and *ycgZ*-*ymgABC* operon expression.

## Materials and methods

### Construction of plasmids and strains

The *pprM* gene (*dr0907*) was PCR amplified from *D. radiodurans* R1 (ATCC13939) genomic DNA using pprM-F and pprM-R primers (Table S1). The amplified product was digested with *Bsa*I and ligated into the *Bsa*I site of the pASK-IBA3plus plasmid (IBA, Germany), which carries the inducible tetracycline promoter/operator for the regulated expression of proteins. The resulting plasmid, named pPprM, was transformed into *E. coli* EPI300 cells (F^−^ λ^−^ *mcrA* Δ(*mrr*-*hsdRMS*-*mcrBC*) φ80d*lacZ*ΔM15 Δ*lacX*74 *recA1 endA1 araD139* Δ(*ara, leu*)*7697 galU galK rpsL*(Str^R^) *nup*G trfA dhfr) (Epicentre, USA) to generate the Ec-PprM strain. The *E. coli* strain carrying the empty vector, pASK-IBA3, was designated as Ec-pASK. The *E. coli ycgZ, ymgA, ymgB*, and *ymgC* genes were PCR amplified using forward and reverse primers specific for each gene, as detailed in Table S1. The entire *ycgZ*-*ymgABC* operon was PCR amplified using ycgZ-F and ymgC-R primers (Table S1). Each PCR product was cloned into *Eco*RI and *Pst*I sites in pASK-IBA3. The resulting plasmids, pYcgZ, pYmgA, pYmgB, pYmgC, and pYZYC (carrying the entire *ycgZ*-*ymgABC* operon) were verified through nucleotide sequencing and transformed into EPI300. To construct the *E. coli oxyR* mutant strain, a one-step gene inactivation method (i.e., λ-Red recombination system) was used (Datsenko and Wanner, 2000). Briefly, the RED helper plasmid, pKD46, was transformed into EPI300 to generate EPI300-pKD46. A chloramphenicol cassette from pKD3 was PCR amplified using oxyR-MF and oxyR-MR primers (Table S1), and the resulting PCR product was transformed into EPI300-pKD46 through electroporation. The *oxyR* mutation was confirmed by PCR using the diagnostic primers, oxyR-DF and oxyR-DR (Table S1), followed by nucleotide sequencing.

### Growth conditions

The *E. coli* recombinant strains carrying pASK-IBA3 and its derivatives were routinely cultivated in LB medium (1% tryptone, 0.5% yeast extract, and 0.5% NaCl) at 37°C with shaking or on LB agar supplemented with 1.5% Bacto-agar. A stationary-phase culture grown for 18 h was used as the seed culture. The seed culture was inoculated into fresh LB broth at a 1:100 dilution and grown to mid-log phase (OD_600_ ≈ 0.5) at 37°C. For protein expression, the mid-log cultures of *E. coli* were further incubated for 2 h in the presence of anhydrotetracycline (AHT), an inducer of the tetracycline promoter of pASK-IBA vectors. Ampicillin (100 μg/ml) and chloramphenicol (25 μg/ml) were used when necessary.

### Preparation of cell-free extracts

After centrifugation, the cell pellet was resuspended in lysis buffer [50 mM Tris-HCl (pH 8.0), 300 mM NaCl, 4 mM 2-mercaptoethaol, 1 mM phenylmethylsulfonyl fluoride (PMSF) and 10% glycerol] and lysed using a VP-15S homogenizer (TAITEC, Japan). The cell lysates were subjected to centrifugation at 12,000 *g* for 20 min at 4°C. The protein concentration in the clarified supernatant was determined using the Bradford colorimetric assay with bovine serum albumin (BSA) as the standard.

### Western blot analysis

The *pprM* gene was PCR amplified using pprM-WF and pprM-WR primers (Table S1) and subsequently cloned into the *Nde*I and *Xho*I sites of the pET-22b expression vector. The resulting plasmid was used to transform *E. coli* BL21 (DE3). When the recombinant strain reached an OD_600_ of 0.5 at 30°C, 1 mM isopropyl β-D-1-thiogalactopyranoside (IPTG) was added to the cultures. Following PprM expression for 4 h at 30°C, PprM was purified using a Ni-NTA column as previously described (Jeong et al., 2016a). The purified PprM protein was used for immunizing CD-1 mice to obtain a polyclonal antibody against PprM, which was subsequently used in Western blot analysis. Following 2-h incubation with AHT, Ec-pASK and Ec-PprM were harvested for lysis (see “Preparation of cell-free extracts”). Equivalent amounts of cell-free extract (10 μg) were resolved on a 12% Bis-Tris gel with MOPS buffer and subsequently transferred onto a polyvinylidene fluoride (PVDF) membrane. The PVDF membranes were incubated with the primary antibody to PprM (1:5000) and sequentially probed with peroxidase-conjugated goat anti-mouse antibody (1:5000) (Sigma, USA). The secondary antibody was detected using a Pierce ECL Western Blotting Substrate according to the manufacturer's instructions (Thermo Scientific, USA). The chemiluminescent signals on the PVDF membrane were visualized using a ChemiDoc Touch Imaging System (Bio-Rad, USA).

### Survival studies

Survival studies were performed 2 h after the addition of AHT (200 ng/ml). The *E. coli* cultures were harvested, washed, and resuspended in phosphate buffer (20 mM, pH 7.4). The cells were incubated with different concentrations of H_2_O_2_ (ranged from 0 to 40 mM) for 1 h. Acid stress survival studies were performed as described previously (Lee et al., 2007b). The cells was diluted 40-fold in LB acidified to pH 2.5 and incubated at 37°C for different time intervals. The stressed cells were serially diluted with phosphate buffer, and 10 μl of each dilution was spotted on LB-ampicillin agar plates. For continuous exposure to H_2_O_2_, the cells were adjusted to give ~10^7^ CFU/ml (OD_600_ ≈ 0.1) and then serially diluted 10-fold in phosphate buffer from 10^7^ to 10^2^ CFU/ml. A 10-μl volume (10^5^ to 10^0^ CFU) from each diluted suspension was spotted on the LB agar plates containing H_2_O_2_ (ranged from 0 to 0.6 mM). The plates were incubated at 37°C for 18 h prior to the enumeration of the colonies. The survival fraction was calculated by dividing the number of colonies of stressed cells by the number of colonies of non-stressed cells.

### Quantitative real-time PCR (qRT-PCR)

Following the 2 h incubation with AHT (200 ng/ml), the cells were exposed to 10 mM of H_2_O_2_ for 15 min and immediately mixed with 10% volume (v/v) of ice-cold phenol-ethanol mixture (5% phenol and 95% ethanol) to preserve RNA integrity. Total RNA was isolated both from non-stressed and stressed cells using a RiboEX reagent (GeneAll, Korea), treated with DNase, and purified using a RNeasy Mini Kit (Qiagen, Germany) according to the manufacturer's instructions. cDNA was synthesized from 1 μg of RNA from each of the samples using a PrimeScript 1st strand cDNA Synthesis Kit (Takara Bio, Japan). For qRT-PCR analysis, 20 μl of the reaction solution containing 10 μl of 2 × SYBR Green PCR Mix (Takara Bio), 1 μl of template cDNA, 0.5 μl of each primer, and 8 μl of RNase-free water was prepared. The housekeeping *gap* gene encoding glyceraldehyde-3-phosphate dehydrogenase (GAPDH) was used as an internal control. The qRT-PCR was run at 95°C for 10 min, followed by 40 cycles of 95°C for 15 s, 60°C for 15 s, and 72°C for 15 s. Amplification, data acquisition, and analysis were performed on an Eco™ Real-Time PCR System (Illumina, USA). Data expressed as means ± standard errors were compared for statistical significance by unpaired *t*-test. The primer sequences used for qRT-PCR are listed in Table S2.

### Catalase activity assay

The cell-free extracts were prepared as mentioned in “Preparation of cell extracts.” Catalase activity was measured using a spectrophotometric assay as previously described (Jeong et al., 2016a). Ten micrograms of the cell extract were mixed with 1 ml of 50 mM phosphate buffer (pH 7.0) containing 10 mM H_2_O_2_ and incubated for 5 min at 25°C. Catalase activity was calculated from the rate of decrease in absorbance at 240 nm resulting from the decomposition of H_2_O_2_. After the rate of decrease in the absorbance was calculated, the catalase activities were expressed as a percentage relative to wild type (set to 100%).

### Determination of intracellular Mn/Fe ratio

Total amounts of intracellular iron and manganese were measured using an Inductively Coupled Plasma Atomic Emission Spectrometer (ICP-AES). Following 2 h incubation with AHT (200 ng/ml), one-liter cultures of Ec-PprM and Ec-pASK strains were centrifuged and washed twice with ice-cold 10 mM Tris-HCl containing 1 mM EDTA and once with ice-cold 10 mM Tris-HCl. After lyophilization, the metal contents in the samples were determined using an Optima 4300 DV ICP spectrometer (Perkin Elmer, USA) at the Korea Basic Science Institute (KBSI, Gwangju, South Korea).

### EPR spin trapping

The amounts of hydroxyl radical (·OH) in Ec-PprM and Ec-pASK cells were detected using electron paramagnetic resonance (EPR) spectroscopy with the specific probe ethanol/a-(4-pyridyl-1-oxide)-N-tert-butylnitrone (4-POBN) as previously described (Lee et al., 2009). Following 2 h incubation with AHT (200 ng/ml), the cells were washed twice with ice-cold Chelex-treated Hank's balanced salt solution (HBSS) and resuspended in ice-cold HBSS. The cell suspension was added to a mixture containing 100 mM diethylenetriaminepentaacetic acid (DTPA), 10 mM 4-POBN, 170 mM ethanol, 1 mM H_2_O_2_, and HBSS. The final cell densities were equivalent in all samples (OD_600_ of ~20). The EPR spectra were measured on a JES-FA200 ESR spectrometer (JEOL, Japan). The spectra were recorded at room temperature with a 9.4 GHz microwave frequency, 10 mW microwave power, 0.2 mT modulation amplitude, 100 kHz modulation frequency, and 3.0 × 10 amplification.

### Detection of carbonylated proteins

Following the 2 h incubation with AHT (200 ng/ml), the cells were exposed to 10 and 20 mM H_2_O_2_ for 1 h. The cell-free extracts (50 μg) were prepared as mentioned in “Preparation of cell extracts,” and protein carbonylation was detected as previously described (Daly et al., 2007). The carbonyl groups in the *E. coli* protein samples were derivatized to 2,4-dinitrophenylhydrazone by reaction with 2,4-dinitrophenylhydrazine (DNPH) using a OxyBlot Protein Oxidation Detection Kit (Millipore, USA) according to the manufacturer's instructions. The protein samples were resolved on a 12% Bis-Tris gel and subsequently transferred to PVDF membrane. The dinitrophenol (DNP) adduct of the carbonyls were probed using the primary and secondary antibodies provided in the Oxyblot Kit (Millipore). The chemiluminescent signals produced using the Pierce ECL Western Blotting Substrate were visualized using the ChemiDoc Touch Imaging System (Bio-Rad).

## Results

### Overexpression of *pprM* in *E. coli* enhances H_2_O_2_ tolerance

**Figure 1:**
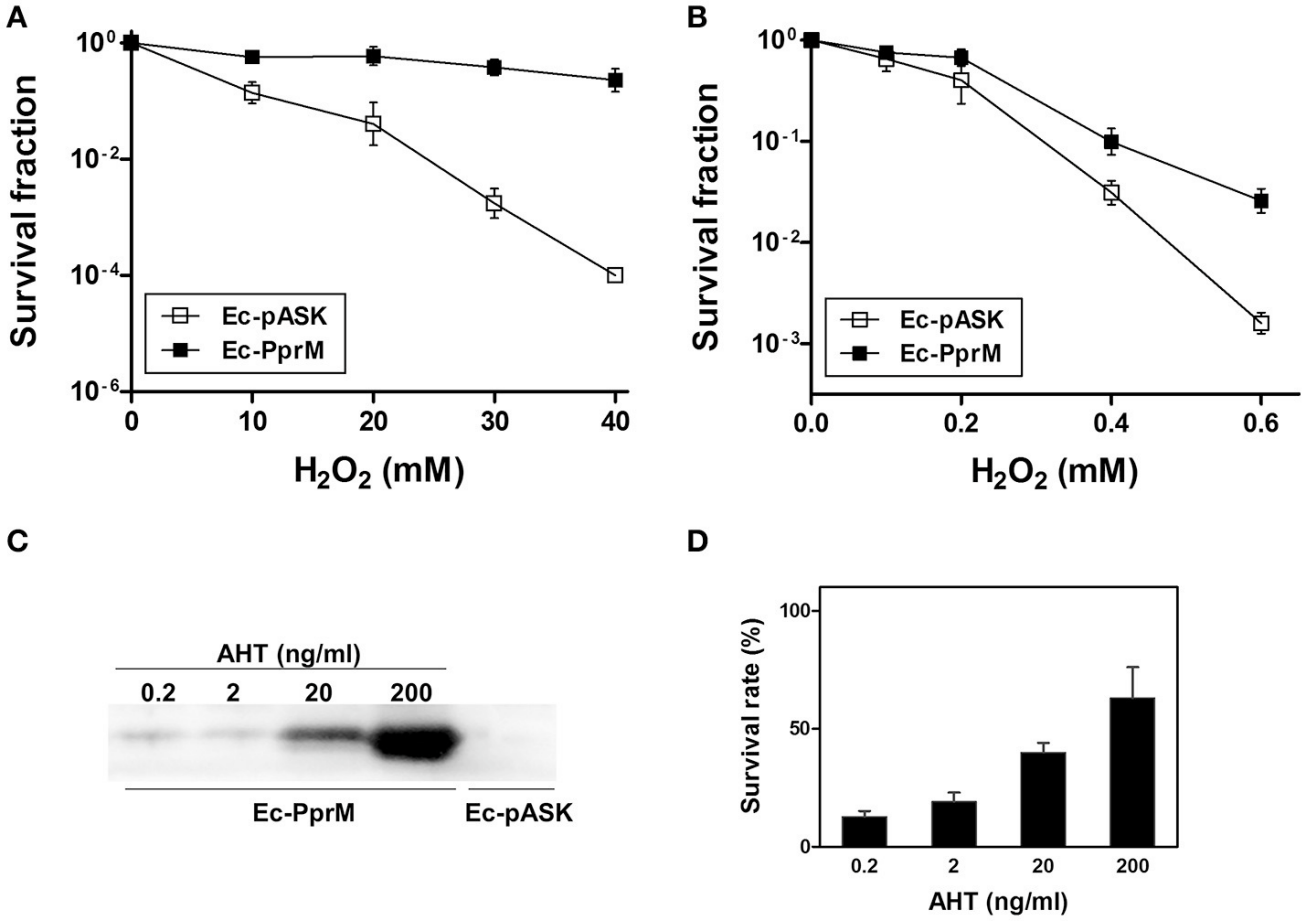
**Survival of ***E. coli*** expressing ***pprM*** under H_**2**_O_**2**_ stress**. Following the 2 h incubation with AHT (200 ng/ml), cells expressing *pprM* (Ec-PprM) and carrying an empty vector (Ec-pASK) were exposed to H_2_O_2_ (10, 20, 30, and 40 mM) for 1 h **(A)** or were spotted on the plates containing H_2_O_2_ (0.1, 0.2, 0.4, and 0.6 mM) **(B)**. The survival fraction was calculated by dividing the colony-forming units (CFUs) of H_2_O_2_-treated cells by the CFUs of non-treated cells. The error bars represent the standard deviation of three independent experiments (*n* = 3). **(C)** Ec-PprM grown to early log phase was incubated at the indicated concentrations of AHT for 2 h, and Ec-pASK was incubated in 200 ng/ml AHT. Equivalent amounts of cell-free extract (10 μg) were loaded in each lane. The PprM levels were measured through Western blotting using anti-PprM antibodies. **(D)** Ec-PprM was incubated in different concentrations of AHT for 2 h and subsequently exposed to 20 mM H_2_O_2_ for 1 h. The survival rate was measured through comparison to non-treated cells, with the CFUs of non-treated cells set to 100%. The error bars represent the standard deviation of three independent experiments (*n* = 3).

To determine whether PprM protects *E. coli* cells from oxidative stress, *E. coli* cells expressing PprM (Ec-PprM) or carrying an empty vector (Ec-pASK) were grown to early log phase, followed by incubation for 2 h with anhydrotetracycline (AHT) to produce PprM. Subsequently, these cells were treated with different amounts of H_2_O_2_ (0–40 mM) for 1 h. The surviving fraction of Ec-pASK was dramatically decreased in a H_2_O_2_ concentration-dependent manner, whereas Ec-PprM maintained the survival rate (Figure 1A). The incubation of Ec-pASK with 40 mM of H_2_O_2_ decreased cell survival for approximately 4 log cycles, but Ec-PprM easily survived incubation with up to 40 mM of H_2_O_2_ with less than 1 log cycle of reduction (Figure 1A). Next, to determine the PprM effect at low concentration of H_2_O_2_ (below 1 mM), Ec-PprM cells were spotted on the plates containing H_2_O_2_ and incubated for an extended period of time (18 h) because exposure time is inversely related to concentration. The survival fraction of Ec-PprM grown in the presence of H_2_O_2_ was reduced but still was 0.5 and 1-log cycle higher at 0.4 and 0.6 mM H_2_O_2_, respectively, relative to Ec-pASK (Figure 1B). To investigate the relevance of H_2_O_2_ tolerance according to the PprM expression level, we monitored the cell survival rate under different AHT concentrations, followed by treatment with 20 mM H_2_O_2_. Western blot analysis showed that the cellular levels of PprM protein were increased in an AHT concentration-dependent manner (Figure 1C), and cell survival was also gradually increased in a PprM dependent-manner (Figure 1D). Taken together, these results indicate that the heterologous expression of PprM enhances the oxidative stress tolerance of *E. coli*, and the level of tolerance is directly associated with the amount of PprM in the cell.

### Oxidative stress response genes are activated in Ec-PprM

**Figure 2:**
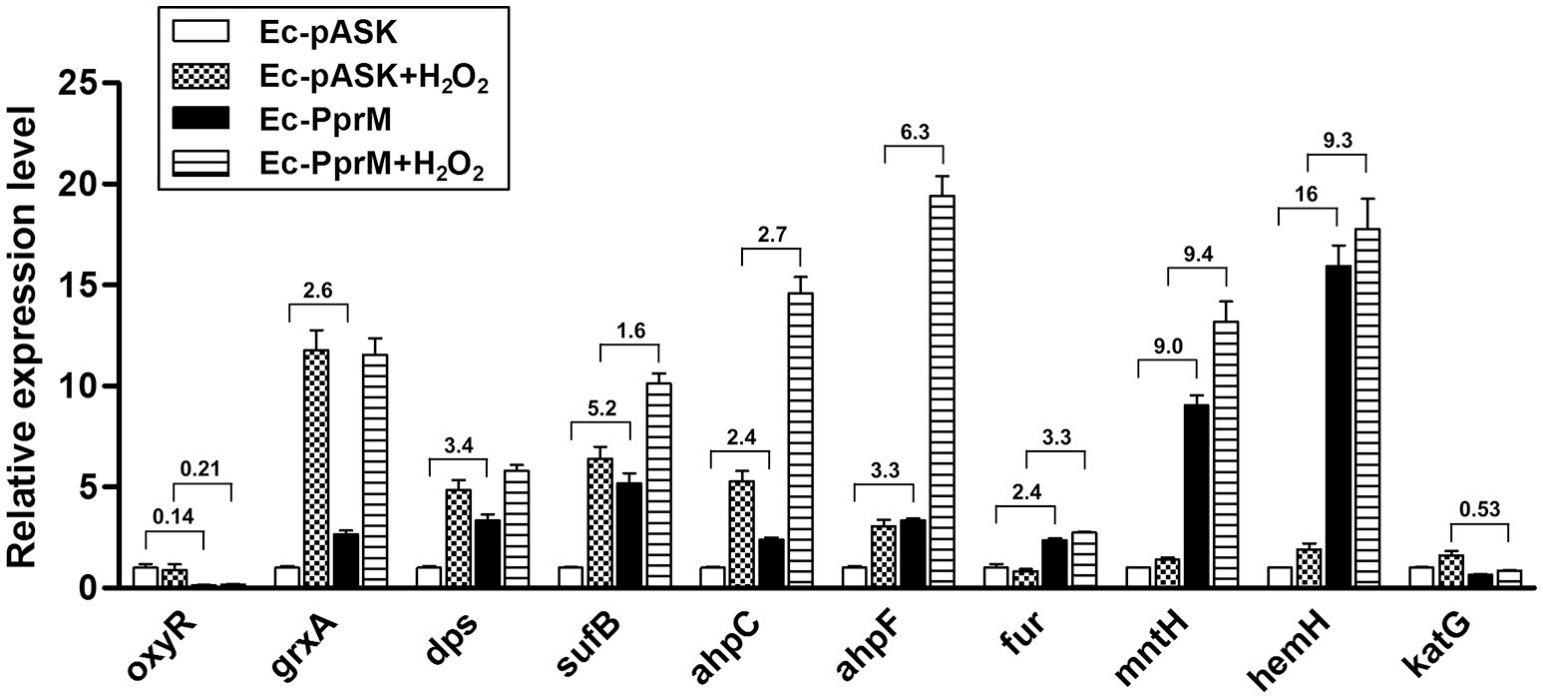
**qRT-PCR assay of OxyR-dependent genes in Ec-PprM**. Following the 2 h incubation with AHT (200 ng/ml), cells were exposed to 10 mM H_2_O_2_ for 15 min. The relative expression values were determined by defining the mRNA levels of each gene from non-treated Ec-pASK cells as 1. The error bars represent the standard error of three independent experiments conducted in duplicate (*n* = 3). Fold changes of genes showing a statistically significant difference (*p* < 0.05) in expression between Ec-pASK and Ec-PprM are indicated.

The OxyR transcription factor is one of the major regulators activated during oxidative stress in *E. coli*. OxyR immediately senses the presence of H_2_O_2_ and induces the antioxidant system (Chiang and Schellhorn, 2012). To examine the mechanism of how PprM confers H_2_O_2_ tolerance to *E. coli*, we initially determined the mRNA levels of OxyR-dependent genes, including *grxA* (glutaredoxin 1), *dps* (nonspecific DNA binding protein), *sufB* (Fe-S cluster assembly protein), *ahpCF* (alkyl hydroperoxide reductase), *fur* (ferric uptake regulator), *mntH* (Mn transporter), *hemH* (ferrochelatase), and *katG* (bifunctional hydroperoxidase I, HPI) using quantitative RT-PCR (qRT-PCR) analysis. Although the expression of *oxyR* was down-regulated, we observed that most of the OxyR-dependent genes tested, except *katG*, were increased in Ec-PprM prior to H_2_O_2_ treatment compared with Ec-pASK (Figure 2). Thus, it is likely that the pre-activation of these genes prepares the cells for oxidative stress by increasing their antioxidant capacity prior to H_2_O_2_ exposure. When the gene expression levels were compared between Ec-PprM and Ec-pASK cells treated with H_2_O_2_, the expression levels of *sufB, ahpCF, fur, mntH*, and *hemH* were higher in Ec-PprM+H_2_O_2_ than in Ec-pASK+H_2_O_2_, and *grxA* and *dps* expressions were comparable in both strains (Figure 2). Particularly, the fold increases (from 9- to 16-fold) of *mntH* and *hemH* expression were much higher than other OxyR-dependent genes tested, regardless of H_2_O_2_ treatment (Figure 2). These results suggest that *mntH* and *hemH* play more important roles than other genes in conferring H_2_O_2_ tolerance to Ec-PprM.

### Increased intracellular manganese protects proteins in Ec-PprM

**Figure 3:**
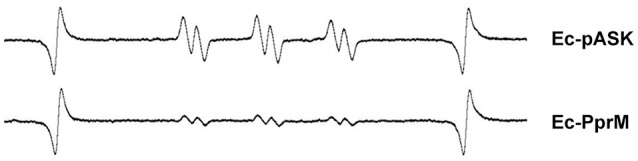
**EPR absorption of the POBN-hydroxyethyl adduct diagnostic for ·OH**. Following the 2 h incubation with AHT (200 ng/ml), the cell suspension of Ec-pASK and Ec-PprM was mixed with 4-POBN and ethanol. The presence of POBN-adducts was detected using EPR spectroscopy.

The modulation of the cellular metal ion content is an important and widespread component of bacterial adaptive responses to H_2_O_2_. Particularly, a high manganese to iron (Mn/Fe) ratio has been correlated with ROS resistance (Faulkner and Helmann, 2011). One of the distinct features observed in the qRT-PCR results was the up-regulation of *mntH*, which encodes the Mn transporter (Figure 2). Because the enhanced expression of *mntH* might increase the intracellular Mn concentration, we compared the Mn concentration between Ec-pASK and Ec-PprM. Ec-PprM showed more than 2 times higher Mn levels than Ec-pASK, and the Fe level decreased by approximately 25% in Ec-PprM compared with Ec-pASK. Notably, the Mn/Fe ratio was at least 2.5 times higher in Ec-PprM (Table 1). In a spontaneous reaction catalyzed by ferrous iron (Fenton reaction), H_2_O_2_ can be converted to highly reactive ·OH (Lisher and Giedroc, 2013). Intracellular ·OH is trapped by ethanol/4-POBN, forming hydroxyethyl-POBN adducts, which can be detected by electron paramagnetic resonance (EPR) spin trapping (Lee et al., 2009). The relative intensity of the six-line EPR signal, which is characteristically exhibited by POBN radical adducts, was markedly decreased in Ec-PprM compared with Ec-pASK (Figure 3), demonstrating that the ·OH level is lower in Ec-PprM than in Ec-pASK. This result is consistent with the decreased Fe level in Ec-PprM (Table 1).

**Table 1:** **Intracellular Mn and Fe contents^**a**^**.

**1st experiment**						**2nd experiment**					
**Ec-pASK**			**Ec-PprM**			**Ec-pASK**			**Ec-PprM**		
**Mn**	**Fe**	**Mn/Fe**	**Mn**	**Fe**	**Mn/Fe**	**Mn**	**Fe**	**Mn/Fe**	**Mn**	**Fe**	**Mn/Fe**
0.12^b^	3.75	0.032	0.27	2.89	0.093	0.11	4.53	0.029	0.24	3.15	0.076

a Two independent experiments were carried out as described in “Materials and Methods.”

b *The metal content was normalized to the dry weight (nmol/mg cell)*.

**Figure 4:**
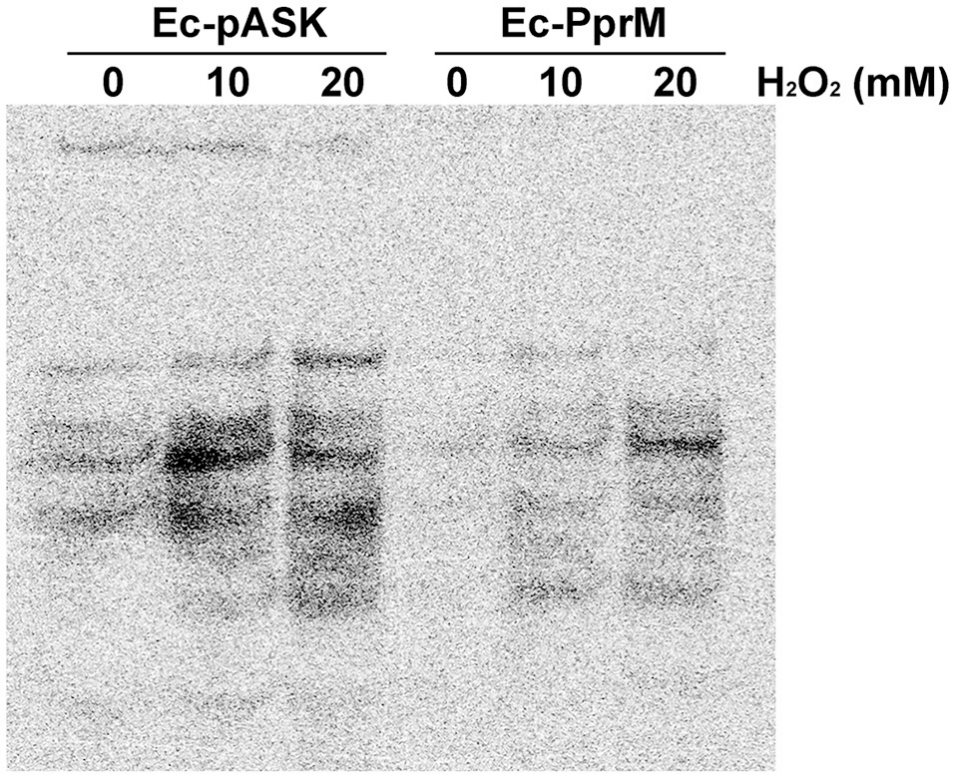
**Protein carbonylation assay of Ec-PprM**. Following the 2 h incubation with AHT (200 ng/ml), cells were exposed to the indicated concentrations of H_2_O_2_ for 1 h. Equivalent amounts of cell-free extract (50 μg) were loaded in each lane. The protein carbonyl groups (a marker of oxidative damage) were derivatized with DNPH and detected using an antibody to DNP.

Bacteria with high Mn/Fe ratios are resistant to protein oxidation, whereas bacteria with low Mn/Fe ratios are sensitive to protein oxidation (Daly, 2009). The level of carbonyl groups in proteins, detected through Western blot analysis, has been widely used as a marker of oxidative protein damage (Daly et al., 2007). The proteins extracted from Ec-PprM exhibited substantially less carbonyl groups compared with Ec-pASK (Figure 4). After H_2_O_2_ treatments, high levels of protein oxidation occurred in Ec-pASK, whereas low levels of protein carbonylation were observed in Ec-PprM, suggesting that the high Mn/Fe ratio in Ec-PprM renders *E. coli* cells more tolerant to oxidative stress through the protection of proteins from ROS.

### Ec-PprM shows H_2_O_2_ tolerance in the absence of *oxyR*

**Figure 5:**
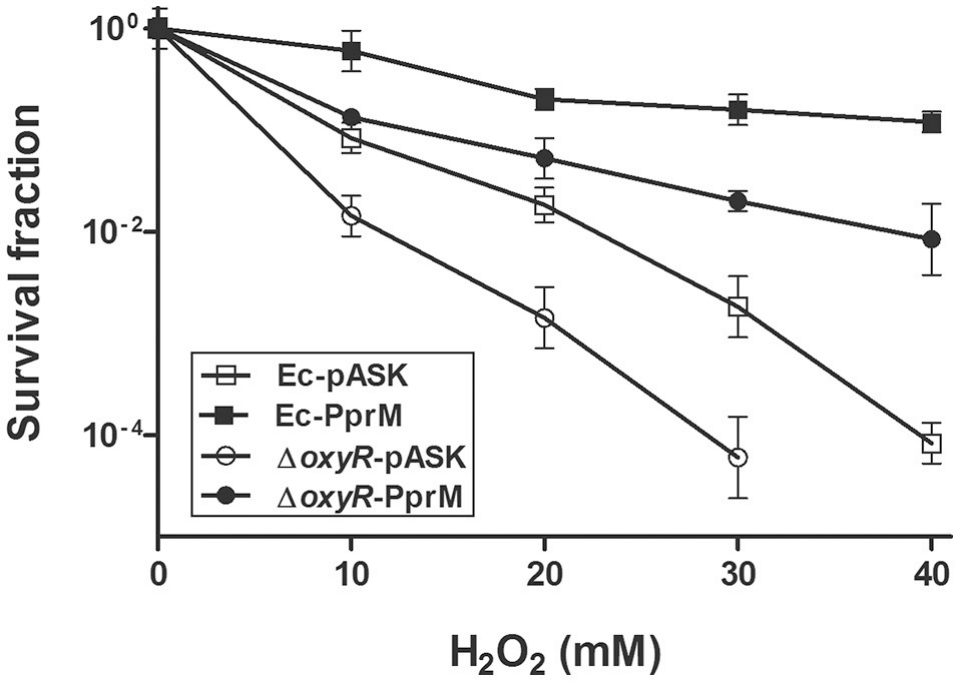
**Survival of ***oxyR*** mutants under H_**2**_O_**2**_ stress**. Following the 2 h incubation with AHT (200 ng/ml), the wild-type and *oxyR* mutant strains expressing *pprM* (Ec-PprM and Δ*oxyR*-PprM, respectively) and carrying an empty vector (Ec-pASK and Δ*oxyR*-pASK, respectively) were exposed to H_2_O_2_ at the indicated concentrations for 1 h. The survival fraction was calculated by dividing the CFUs of H_2_O_2_-treated cells by the CFUs of non-treated cells. The error bars represent the standard deviation of three independent experiments (*n* = 3).

Because *oxyR* expression was not induced in Ec-PprM (Figure 2), an *E. coli oxyR* mutant strain was constructed to investigate whether OxyR is necessary for the enhanced H_2_O_2_ tolerance of Ec-PprM. As expected, *E. coli* cells became sensitive to H_2_O_2_ stress in the absence of *oxyR*. The *oxyR* mutant strain carrying an empty vector (Δ*oxyR*-pASK) could not survive under 40 mM H_2_O_2_ (Figure 5). Interestingly, when *pprM* was expressed in *oxyR* mutants (Δ*oxyR*-PprM), Δ*oxyR*-PprM showed a greater H_2_O_2_ tolerance than Ec-pASK. The survival rate of Δ*oxyR*-PprM was approximately 2 log cycles higher than that of Ec-pASK under 40 mM H_2_O_2_ (Figure 5). In addition, the expression of the OxyR-dependent genes (*sufB, dps*, and *mntH*) tested was not increased in Δ*oxyR*-PprM (data not shown). These results suggest that OxyR-independent genes contribute to PprM-mediated H_2_O_2_ tolerance in the *oxyR* deletion background.

### Activation of *ycgZ*-*ymgABC* leads to elevated H_2_O_2_ tolerance

**Figure 6:**
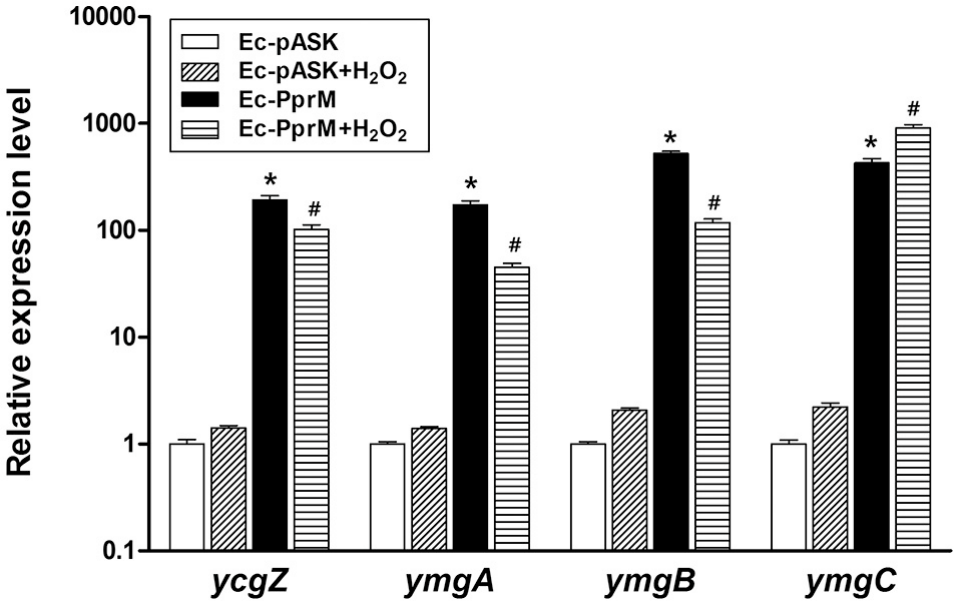
**qRT-PCR assay of the ***ycgZ***-***ymgABC*** operon**. Following the 2 h incubation with AHT (200 ng/ml), cells were exposed to 10 mM H_2_O_2_ for 15 min. The relative expression values were determined by defining the mRNA levels of each gene from non-treated Ec-pASK cells as 1. The error bars represent the standard error of three independent experiments conducted in duplicate (*n* = 3). Data were analyzed by Student's *t*-test. ^*^p < 0.01 compared with Ec-pASK; ^#^*p* < 0.01 compared Ec-pASK+H_2_O_2_.

**Figure 7:**
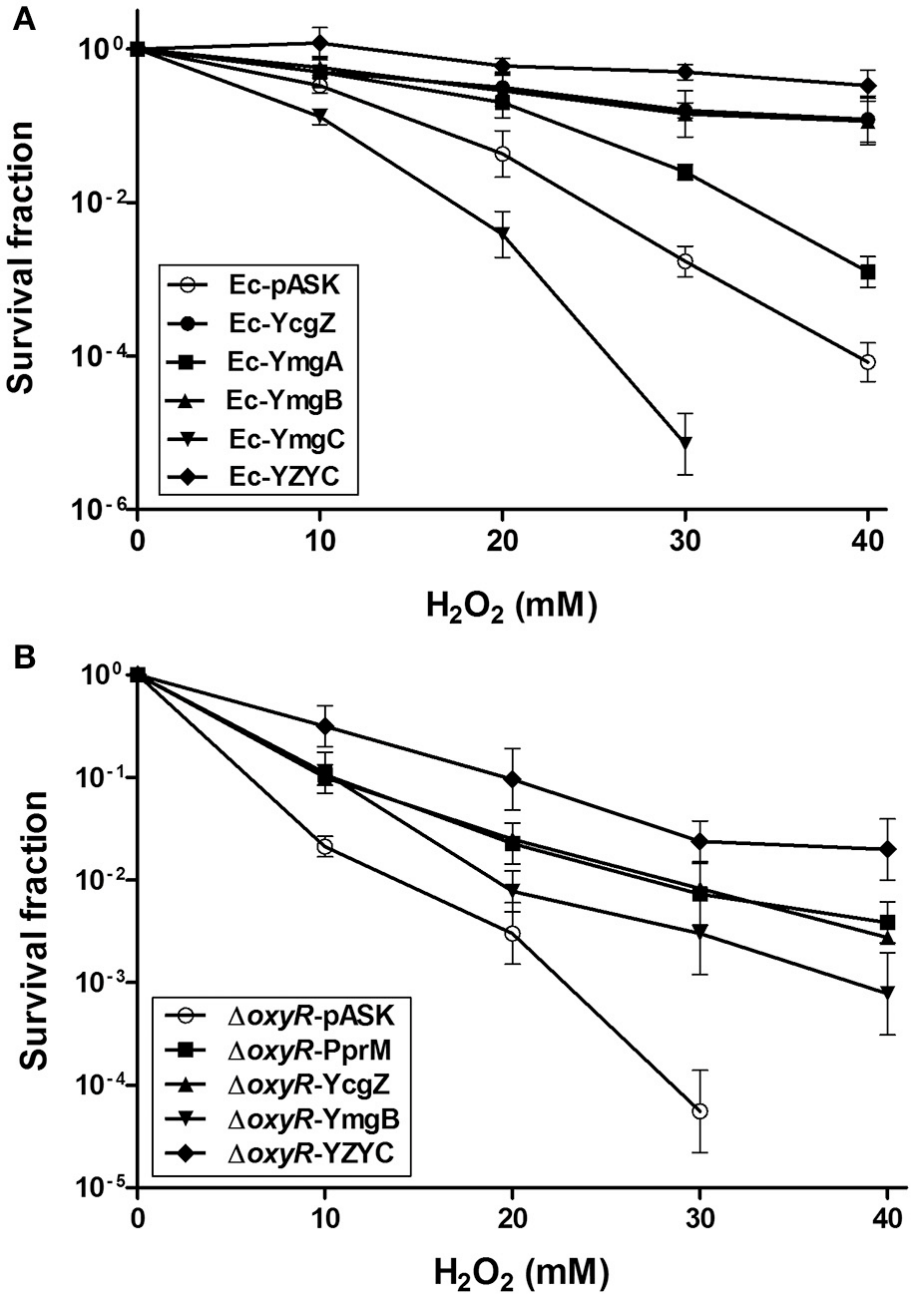
**Survival of ***E. coli*** expressing the ***ycgZ*** and ***ymg*** genes under H_**2**_O_**2**_ stress**. Following the 2 h incubation with AHT (200 ng/ml), the recombinant strains were exposed to H_2_O_2_ at the indicated concentrations for 1 h. The survival fraction was calculated by dividing the CFUs of H_2_O_2_-treated cells by the CFUs of non-treated cells. The error bars represent the standard deviation of three independent experiments (*n* = 3). **(A)** The wild-type strains expressing *ycgZ, ymgA, ymgB, ymgC*, and the entire *ycgZ*-*ymgABC* operon were designated Ec-YcgZ, Ec-YmgA, Ec-YmgB, Ec-YmgC, and Ec-YZYC, respectively. **(B)** The *oxyR* mutant strains expressing *pprM, ycgZ, ymgB*, and the entire *ycgZ*-*ymgABC* operon were designated Δ*oxyR*-PprM, Δ*oxyR*-ycgZ, Δ*oxyR*-YmgB, and Δ*oxyR*-YZYC, respectively.

A previous study has reported the activation of a set of genes by H_2_O_2_ in Δ*oxyR* (Zheng et al., 2001). Some of these genes were elevated in Ec-PprM in the absence of H_2_O_2_ stress (Figure S1). Among the 29 genes tested, *ymgA* and *ymgB* showed the highest expression levels (Figure S1). In *E. coli, ycgZ, ymgA, ymgB*, and *ymgC* constitute an operon (*ycgZ*-*ymgABC*) that encodes small proteins of 78–90 amino acids (Tschowri et al., 2009, 2012). We confirmed that all four genes were expressed more than 100-fold in Ec-PprM, regardless of H_2_O_2_ treatments (Figure 6). Next, we constructed *E. coli* strains overexpressing each gene of the *ycgZ-ymgABC* operon and examined the viability of strains subjected to H_2_O_2_ stress. Ec-YcgZ and Ec-YmgB showed robust H_2_O_2_ stress tolerance (Figure 7A): the survival rates of both strains were approximately 3 log cycles higher than that of Ec-pASK at 40 mM H_2_O_2_, which is comparable to that of Ec-PprM (Figure 1A). Ec-YmgA was less tolerant to H_2_O_2_ than Ec-YcgZ and Ec-YmgB but still showed an approximately 1-log cycle increase in the survival rate compared with Ec-pASK at 30 and 40 mM H_2_O_2_ (Figure 7A). Although Ec-YmgC was more sensitive to H_2_O_2_ than Ec-pASK, the *E. coli* strain overexpressing the *ycgZ-ymgABC* operon (Ec-YZYC) exhibited the highest H_2_O_2_ stress tolerance (Figure 7A). The cell survival rate of the *E. coli* strain overexpressing the *ycgZ-ymgAB* region was not different from that of Ec-YZYC (data not shown), suggesting that the negative effect of YmgC overexpression on cell survival can be masked by the simultaneous expression of the *ycgZ, ymgA*, and *ymgB* genes in Ec-YZYC. The overexpression of the entire operon markedly increased the cell survival rate of Δ*oxyR* under H_2_O_2_ stress conditions, which was higher than those of not only Δ*oxyR*-YcgZ and Δ*oxyR*-YmgB but also Δ*oxyR*-PprM (Figure 7B). These results suggest that the activation of the *ycgZ*-*ymgABC* operon through PprM enhances the H_2_O_2_ stress tolerance of *E. coli* in the absence of OxyR.

## Discussion

Stress tolerance and cellular robustness are key characteristics for industrial microbes. In an effort to improve the robustness of *E. coli* as an industrial host, many strategies have been used, including stress-induced mutagenesis, combinatorial gene deletion, engineering of transcriptional regulators, and synthetic biology (Zhu et al., 2012; Lin et al., 2013; Lennen and Herrgård, 2014; Zhu et al., 2014). The introduction or overexpression of specific proteins, such as heat shock proteins (HSPs), also enhances resistance at the cellular level (Jia et al., 2014). Particularly, microbes living in extreme environments, known as extremophiles, provide genetic resources for developing robust hosts (Lin et al., 2013; Jia et al., 2014; Appukuttan et al., 2016). Here, we enhanced the oxidative stress (H_2_O_2_) tolerance of *E. coli* through the heterologous expression of PprM, a CSP homolog of the extraordinary γ-radiation-resistant bacterium *D. radiodurans* (Figure 1).

The *E, coli* OxyR transcription factor is one of the major regulators activated during oxidative stress. OxyR controls a regulon of approximately 40 genes induced through H_2_O_2_ and protects *E. coli* from H_2_O_2_ toxicity. Upon exposure to H_2_O_2_, OxyR is activated by the formation of an intramolecular disulfide bond that induces a conformational change, and the oxidized OxyR binds to the promoter regions of target genes to activate transcription (Chiang and Schellhorn, 2012). Although most of the OxyR regulon genes examined in this study were activated in Ec-PprM, *oxyR* transcription was decreased (Figure 2). Because OxyR acts as a repressor of its own transcription (Pomposiello and Demple, 2001), it is likely that OxyR oxidation occurs in Ec-PprM, thereby activating OxyR regulon genes and repressing *oxyR* expression. The thioredoxin (Trx) system and the glutathione (GSH)-glutaredoxin system can deactivate OxyR through the reduction of disulfide bonds in OxyR (Lu and Holmgren, 2014). During aerobic growth, the disruption of both GSH metabolism and Trx partially activates OxyR in the absence of added H_2_O_2_ (Pomposiello and Demple, 2001), and *E. coli* strains without Trx proteins are more resistant to oxidative stress, reflecting the defective OxyR reduction (Lu and Holmgren, 2014), suggesting that multiple systems contribute to the maintenance of OxyR in the reduced state. There is no direct evidence that PprM participates in OxyR activation, but the OxyR reduction systems might be hampered as a result of PprM overexpression.

Catalases protect cells through catalysis of H_2_O_2_ decomposition into oxygen and water (Imlay, 2013). In *E. coli*, deinococcal Ppr proteins PprI and PprA stimulated KatG (bifunctional catalase-peroxidase) and KatE (monofunctional hydroperoxidase II), respectively, thereby improving tolerance against oxidative stress (Gao et al., 2003; Kota and Misra, 2006). However, *katG* was not activated in Ec-PprM (Figure 2), and the total catalase activity of Ec-PprM was not significantly altered following AHT addition (Figure S2). *E. coli* possesses nine CSP homologs, CspA to CspI: CspA, CspB, CspG, and CspI are cold inducible, whereas CspE and CspC are constitutively expressed at physiological temperatures, and CspD is induced under nutrient deprivation (Horn et al., 2007). CSPs function as RNA chaperones that maintain the single-stranded state of RNA by melting the secondary structure for efficient transcription and/or translation (Horn et al., 2007). *katG* requires the melting function of CspE for optimal expression at physiological temperatures (37°C) (Phadtare et al., 2006). PprM also exerted an effect under physiological temperatures (Ohba et al., 2009). Thus, it is likely that *katG* expression might not be activated by the competition of exogenous deinococcal CSP PprM and endogenous *E. coli* CSPs, suggesting that catalase makes a little, if any, contribution to the H_2_O_2_ tolerance of Ec-PprM.

The OxyR regulon contributes to the suppression of the Fenton reaction, and Mn import *via* MntH is a key element of the OxyR response to H_2_O_2_ in *E. coli* (Anjem et al., 2009). *E. coli* is capable of shifting from metabolism based on Fe^2+^ to metabolism based on Mn^2+^ to protect key enzymes from inactivation by ROS (Lisher and Giedroc, 2013). *mntH* was up-regulated by approximately 9-fold in Ec-PprM (Figure 2), and Ec-PprM showed increased intracellular Mn/Fe levels of more than two times compared with wild type (Table 1). Mn acts as a chemical scavenger of ${\text{O}}_{2}^{-}$ through the formation of low molecular weight Mn complexes, such as Mn-phosphate and Mn-carbonate, and metalloenzyme cofactor substitution to prevent Fenton-based protein damage and subsequent enzyme inactivation (Lisher and Giedroc, 2013). Mn has also been implicated in the metalation of the Mn-cofactor superoxide dismutase (SodA), which catalyzes the conversion of ${\text{O}}_{2}^{-}$ to H_2_O_2_ and water (Imlay, 2013). In this context, the low levels of intracellular ·OH and protein oxidation compared with wild type (Figures 3, 4) suggests the possibility that Mn plays an important role in the oxidative stress tolerance of Ec-PprM.

PprM overexpression activated the expression of the *ycgZ*-*ymgABC* operon (Figure 6), which enhanced the H_2_O_2_ stress tolerance of Ec-PprM, even in the absence of OxyR (Figure 7). The *ycgZ*-*ymgABC* operon is located immediately adjacent to the *ycgF*-*ycgE* region separated by a divergently transcribing control region (Tschowri et al., 2009, 2012). The expression of the *ycgZ*-*ymgABC* operon is repressed by YcgE (BluR), and YcgF (BluF) directly antagonizes YcgE, leading to the expression of the *ycgZ*-*ymgABC* operon in a light-dependent manner (Tschowri et al., 2009, 2012). When we examined the expression of *ycgE* and *ycgF*, both genes showed increased expression in Ec-PprM, but the expression of *ycgE* was higher than that of *ycgF* (Figure S1). Thus, the activation of the *ycgZ*-*ymgABC* operon in Ec-PprM might not be dependent on YcgF. It has recently been reported that the promoter region of the *ycgZ*-*ymgABC* operon is bound by *apo*-Fur (iron-free Fur) under iron starvation, and this binding increases transcript levels (Seo et al., 2014). Considering the increased *fur* expression (Figure 2) and decreased Fe levels (Table 1), *apo*-Fur might activate the *ycgZ*-*ymgABC* operon expression in Ec-PprM. Ferrochelatase, encoded by *hemH*, completes heme synthesis through the insertion of a ferrous iron into protoporphyrin IX (Mancini and Imlay, 2015). Notably, *hemH* was markedly induced in Ec-PprM (Figure 2), suggesting the possibility that the increased induction of *hemH* could facilitate *apo*-Fur formation through the reduction of intracellular unincorporated iron. In *E. coli, mntH* transcription is repressed in the presence of iron in a Fur-dependent manner (Patzer and Hantke, 2001), but *mntH* was induced in Ec-PprM, similar to *hemH* (Figure 2). This result suggests that Fur lacked the ferrous iron cofactor and thus did not exert a negative effect. Indole is an extracellular signal in *E. coli*, and this compound represses the *ycgZ*-*ymgABC* operon 2- to 5-fold (Lee et al., 2007a). We observed that the *tnaA* gene, encoding tryptophanase, which converts tryptophan into indole, was repressed approximately 50-fold in Ec-PprM (Figure S1). Further systematic studies are needed to elucidate the mechanisms underlying the up-regulation of the *ycgZ*-*ymgABC* operon in Ec-PprM.

YmgB is important for the survival of *E. coli* against H_2_O_2_ (Lee et al., 2007b), but the molecular functions of the YcgZ and Ymg proteins are not yet clearly understood. Ymg proteins have been implicated in biofilm formation as mutations in *ymgA, ymgB*, and *ymgC* increased biofilm formation (Lee et al., 2007b), and YcgZ partially counteracts the effects of YmgB (Tschowri et al., 2009). However, *ymgC* expression resulted in an effect opposite to that of *ycgZ* and *ymgB* in the present study: the overexpression of *ycgZ* and *ymgB* (and to a lesser extent YmgA) enhanced H_2_O_2_ tolerance, whereas *ymgC* expression decreased H_2_O_2_ tolerance (Figure 7). Of the *ycgZ, ymgA, ymgB*, and *ymgC* mutants, only the *ymgB* mutant showed approximately 40-fold less survival in an acidic LB medium (pH 2.5 for 1 h) (Lee et al., 2007b). Thus, the *E. coli* strains overexpressing the *ycgZ* and/or *ymg* genes were examined for acid stress tolerance in the same conditions (LB medium, pH 2.5). Not only YmgB but also YcgZ rendered *E. coli* more resistant to low-pH stress, and the co-expression of the *ycgZ*-*ymgABC* genes enhanced acid tolerance more than 10-fold compared with Ec-pASK. However, Ec-YmgC showed an approximately 1-log cycle decrease in survival relative to Ec-pASK after 2 h incubation under acidic conditions (Figure S3). Taken together, these results suggest that YmgC might play a different role depending on the types of environmental stressors. Although Northern blot analysis indicated that all four genes are expressed in a single polycistronic mRNA (Tschowri *et al*., 2009), *ycgZ, ymgA*, and *ymgB*, but not *ymgC*, are under the control of the general stress σ factor σ^S^ (RpoS) (Weber et al., 2005). The fact that the *ycgZ*-*ymgAB* region (without *ymgC*) is conserved in various bacteria shows that this region is a functional genetic unit (Tschowri et al., 2012). Of the acid resistance (AR) systems in *E. coli*, AR1 referred to as oxidative system is dependent on σ^S^ (Chung et al., 2006). Because the overexpression of *ycgZ* and/or *ymgAB* expression protected cells from oxidative and acid stress, it is thus possible that the contribution of YcgZ and YmgB to acid stress tolerance lies in the protection against oxidative damage. The investigation of the roles of the YcgZ and Ymg proteins in stress response system sheds light on the stress tolerance mechanisms of not only Ec-PprM but also *E. coli* itself.

Because exposure to low pH leads to oxidative stress (Lund et al., 2014), and increased expression of OxyR-dependent genes are observed by transcriptional profiling of acid tolerant phenotypes (Chung et al., 2006), metabolic and genetic engineering of Ec-PprM for organic acid production can increase productivity at low pH. Ec-PprM can also be applied to the wastewater treatment system like Advanced Oxidation Process (AOP), where high levels of H_2_O_2_ are used as oxidizing agents (Ramteke and Gogate, 2016; Son et al., 2016). The removal efficiency of chemical oxygen demand by the Fenton oxidation-based AOP (FAOP) was significantly enhanced by the addition of 1.5 g/L (approximately 44 mM) of H_2_O_2_ (Ramteke and Gogate, 2016). It has been reported that subsequent biological oxidation using microorganism shows the synergistic effect in oxidizing organic pollutants present in the wastewater pretreated by FAOP (Mandal et al., 2010; Meenatchisundaram et al., 2014; Ramteke and Gogate, 2016). In particular, the efficacy of Fenton processes is enhanced under acidic conditions (pH 2.5 ~ 3.5) (Mandal et al., 2010; Ramteke and Gogate, 2016). Considering the maintained survivability of Ec-PprM at high concentrations of H_2_O_2_ (up to 40 mM) (Figure 1A), in addition to its acid tolerance (Figure S3), it is possible to use this strain in FAOP and to develop a new hybrid FAOP using genetically engineered Ec-PprM.

## Author contributions

SL, YC, and IN contributed with experimental design and results interpretation of this study. SP and HS carried out all experiments, DA and JJ advised the cell survival assay, and SJ purified the PprM protein. SP and HS wrote the draft manuscript, and SL was in charge of writing the manuscript. All authors have made substantial contribution to the work and approved it for publication.

## Funding

This research was supported by Nuclear R&D program of Ministry of Science, ICT & Future Planning (MSIP), Republic of Korea.

### Conflict of interest statement

The authors declare that the research was conducted in the absence of any commercial or financial relationships that could be construed as a potential conflict of interest.
