# Supplementary material for: PprM, a Cold Shock Domain-Containing Protein from Deinococcus radiodurans, Confers Oxidative Stress Tolerance to Escherichia coli
Source: Front Microbiol. 2017 Jan 10;7:2124. doi: 10.3389/fmicb.2016.02124 (PMC5222802; doi:10.3389/fmicb.2016.02124)
Supplement: Supplementary file 1 [file Table1.PDF]

Table S1. List of primers used in construction of plasmid and mutant

Primer	Sequence (5'-3')
Cloning of genes in pASK[a]	
pprM-F	GCGCATGGTCTCTAATGGCAACTGGAAGAGTG
pprM-R	ATATATGGTCTCTGCGCTCCAGCGGTCGTCGCGGC
ycgZ-F	TCTAGAATTCATGCATCAAAATTCAGTGAC
ycgZ-R	TACACTGCAGTTCAAAAAGCAACCCA
ymgA-F	TCTAGAATTCATGAAGACATCTGATAATG
ymgA-R	TCTACTGCAG ATGTATTCTGTTTATTTTC
ymgB-F	TCTAGAATTC ATG CTTGAAGATACTACAA
ymgB-R	TCTACTGCAG CATATCATCAGCTGTGTA
ymgC-F	TCTAGAATTC ATG AATAATTCAATCCCAGAG
ymgC-R	TCTACTGCAGAGAGAGCACGGATTCCCTGT
Cloning of pprM for Western blotting[a]	
pprM-WF	GTTACATATGGCAACTGGAAGAG
pprM-WR	TATACTCGAGCCAGCGGTCGTCGC
Construction of oxyR mutant and diagnostic PCR	
oxyR-MF	TTGCTATTCTACCTATCGCCATGAACTATCGTGGCGATGGAGGATGGATAGTG TAGGCTGGAGCTGCTTC
oxyR-MR	TTAACTACCCGACGATGGCGGAAGCCTATCGGGTAGCTGCGTTAAACGGTCA TATGAATATCCTCCTTAG
oxyR-DF	GTTTCTGTGAGCAATTATCAGTCA
oxyR-DR	GGAACAGAAAGGTGGCGGCAACAC

[a] Restriction enzyme sites are underlined.
