# Supplementary material for: PprM, a Cold Shock Domain-Containing Protein from Deinococcus radiodurans, Confers Oxidative Stress Tolerance to Escherichia coli
Source: Front Microbiol. 2017 Jan 10;7:2124. doi: 10.3389/fmicb.2016.02124 (PMC5222802; doi:10.3389/fmicb.2016.02124)
Supplement: Supplementary file 2 [file Table2.PDF]

Table S2. List of primers used in qRT-PCR assay

Gene	Forward primer (5’-3’)	Reverse primer (5’-3’)
oxyR	GAAGCACAGACCCACCAGTT	CAAACAACGGCACTTCAATG
grxA	TAGATATTCGTGCGGAAGGG	CCGATATGTTGCTGATCGAC
dps	AAACAAGCGCACTGGAACAT	CTGCACTGCACGTTCTGC
sufB	ACTCAGTTTCGGTTGCCACT	GCGGCAAAGAAGTTGTCATT
ahpC	ACTGCAGAAACTGGGCGTAG	GTTGTCGAAGTTACGGGTCAG
ahpF	GTCGAAAGAAGCGCAGTCTC	AATTGCAGTGTGCTTGATGC
fur	GACAACCATCACGTCAGTGC	ACGATACCAGCGTCGTCAA
mntH	ATTTGCACTCCTCGCTCACT	GTAGCCATCATCGCCAGATT
hemH	ACAACGTTTACCGGAGATGC	GTGCCAGTTCATCCCATACC
katG	CTGGTGTGGTTGGTGTTGAG	AGTGACTCGGTGGTGGAAAC
ycgZ	TTGCATACTCAGCAGGAAACTC	GTTCCAGTCGGCAAAGAAGT
ymgA	TCCAGATACTGCGCATGAAG	TGTCAGCTGTCGCTGTTCTT
ymgB	GCAAGTTACTTTCGCAGTTCG	AACATTATCGCCTGAAAGCA
ymgC	AATCTCGAGAGGGAGGTGTTC	TTTAAACGCAAGCGTAAAGC
yfiA	TTGATCAACAAGCTGGAACG	ACGAAGTTGGCGTCTTTCAC
ibpB	CAGCCATTTAGCCTGAGCTT	ATGGGTTCAGGCTCATTACG
ibpA	ACCAGAGCCAGAGTAATGGC	ATTTCCAGTTCGCTCTCAGC
tnaA	TTCCGCATTCGTGTTATTGA	CTTCGCTATCCAGCAGGAAC
yjiD	TCTCCTCATTCGGCATTACC	CTGACGGCAATACCAGCTCT
cysK	TGCACTGGCCTATGTAGCTG	GGTTTGCACCTAACGCTTTC
uxuA	TACCGTAAACAGCATGGCAA	GAAGTAAATACGCGGACCGA
dsdX	TTGCGCTGATGTTGGTTAAA	GCAGTGATAGGGTTGCCAAT
ytfK	GTTATACATCAAGGACGTTGGC	TTGCAGACGCATAACCTGAC
recN	TACAAACGTCTGGCGAACAG	ACTTTGCAGGTTTGCGTCTT
soxS	TACTTGCAACGAATGTTCCG	ACATAACCCAGGTCCATTGC
dsdA	GCACGTTGTTCCTTGGGTAT	CAACACCACACGGCAGATAG
fpr	CAGCGATTGGCCCTTATTTA	TTCCAGTTCCTGCATCAGTG
yeeD	AGCGGCGATGAGTTAGTGAT	GGTGATGGCATGTCCTTCTT
ygaQ	CGGCAAACACTTGAACGTAA	GATCTGCTGGTAGGTGGCAT
yaiA	GCCGATCATCCTAAACCAGA	TCAGGGTCCTCATAGCGTTT
yceP	AGTTGGTCAGACGCTCTGGT	TTACCTGGAAATCACCGGAT
glgS	GTCGCCCGGTCGATATTC	TGACAATACCAGGCATAACGTG
ydcH	TCGCTTTATGTCCTTGTTCG	GACCACTTCCGCATTGTACC
phoH	GGCAGAGGCCCTGATACATA	GAAGCCAAGATCTTCATCGG
ydeN	CTATGTGGCACACGGTGTTT	ATCCTGAGCATCGGTATTGG
sbp	AAGCGACGTCGGTAATCAAC	GAAGTGTACGGTGCGGAGTT
uspF	AACTCAACGCGTGATTAGCC	CGGAATACGCTAAACCCAGA
cysP	CGATAAAGGCAAGCTGATCC	GTTACCCTTACGCACCAGGA
yaeH	GAAATCAGCCCGAATCTACG	TCGTCGAGGATCTTACGCTT
manX	ACCACTAAAGGCGTGCTGTT	GGAATGTTAACGCCTGCAAT
ycgK	TGAAGGCGCTGATACTTACCT	ATATTTGCCAGAAGCAGGGA
ycgE	CAACGCATCGAAGAGATCAA	GGCGAACAGGAAGGTAAACA
ycgF	ATCGCTAAAGGCTGCTGGTA	GGATTCAAGCCACATCCACT
gapA	AGGTCTGATGACCACCGTTC	GGAACGCCATACCAGTCAGT
