# Supplementary material for: PprM, a Cold Shock Domain-Containing Protein from Deinococcus radiodurans, Confers Oxidative Stress Tolerance to Escherichia coli
Source: Front Microbiol. 2017 Jan 10;7:2124. doi: 10.3389/fmicb.2016.02124 (PMC5222802; doi:10.3389/fmicb.2016.02124)
Supplement: Supplementary file 3 [file Image1.PDF]

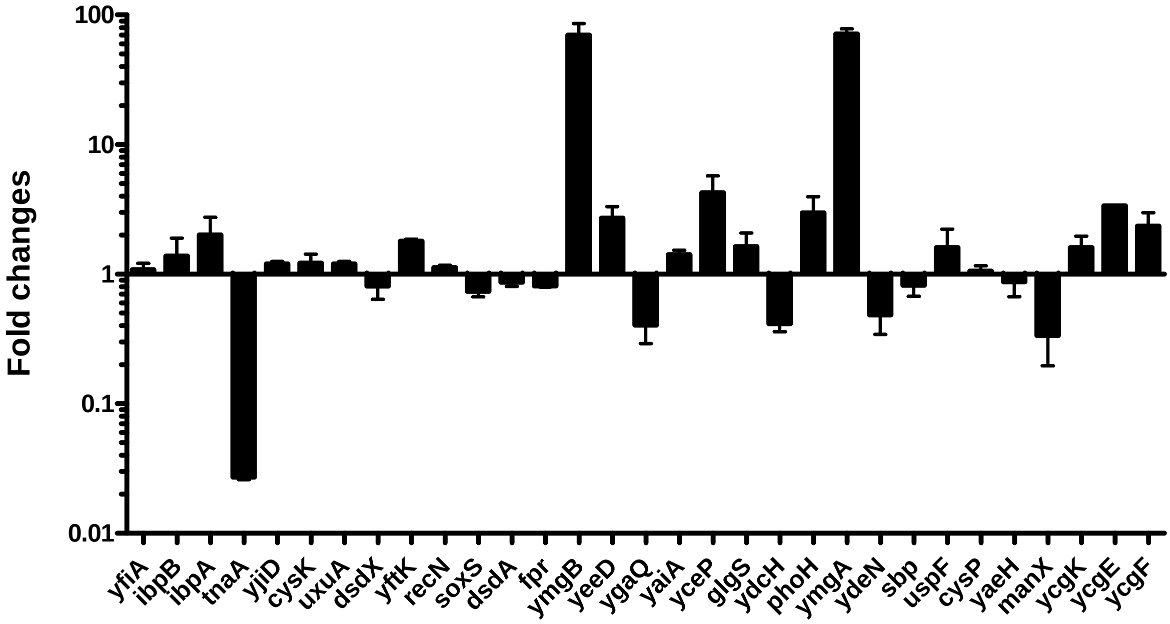

Figure S1. qRT-PCR assay of selected genes in Ec-PprM. The 29 genes (from *yfiA* to *ycgK*), induced independent of OxyR, were selected based on a previous study (Zheng et al., 2001). The last two genes, *ycgE* and *ycgF*, are transcriptional regulators of the *ycgZ-ymgABC* operon (Tschowri et al., 2009). Following the 2 h incubation with AHT (200 ng/ml), total RNA was harvested from Ec-pASK and Ec-PprM and subjected to qRT-PCR to measure the mRNA level of each gene. The fold-changes were obtained by dividing the gene expression levels from Ec-PprM by those from Ec-pASK. The error bars represent the standard error from two independent experiments conducted in duplicate (n=2).
