# Supplementary material for: PprM, a Cold Shock Domain-Containing Protein from Deinococcus radiodurans, Confers Oxidative Stress Tolerance to Escherichia coli
Source: Front Microbiol. 2017 Jan 10;7:2124. doi: 10.3389/fmicb.2016.02124 (PMC5222802; doi:10.3389/fmicb.2016.02124)
Supplement: Supplementary file 4 [file Image2.PDF]

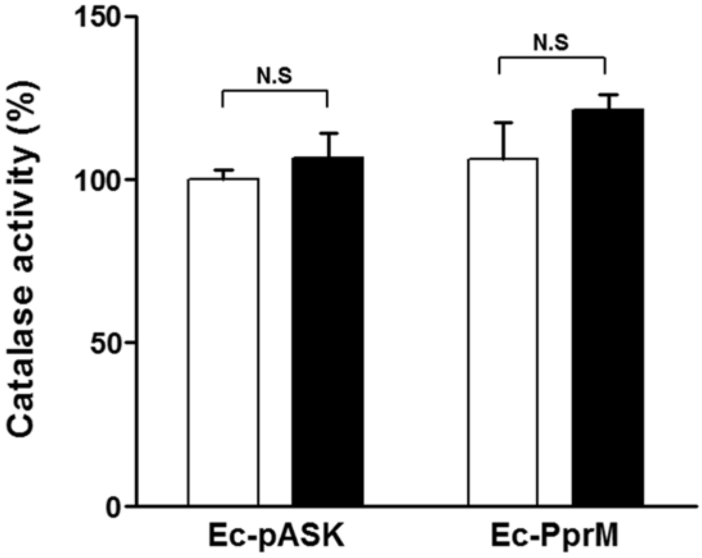

Figure S2. Catalase activity assay. Cells grown to early log phase were further incubated with 200 ng/ml AHT (black bars) and without AHT (white bars) for 2 h. Catalase activities associated with the cell-free extracts of Ec-pASK and Ec-PprM were assessed through spectrophotometric assay. The catalase activity from Ec-pASK incubated without AHT was set to 100%. The error bars represent the standard deviation of three independent experiments (n=3). Data were analyzed by Student's t tests (NS: not significant).
