# Supplementary material for: PprM, a Cold Shock Domain-Containing Protein from Deinococcus radiodurans, Confers Oxidative Stress Tolerance to Escherichia coli
Source: Front Microbiol. 2017 Jan 10;7:2124. doi: 10.3389/fmicb.2016.02124 (PMC5222802; doi:10.3389/fmicb.2016.02124)
Supplement: Supplementary file 5 [file Image3.PDF]

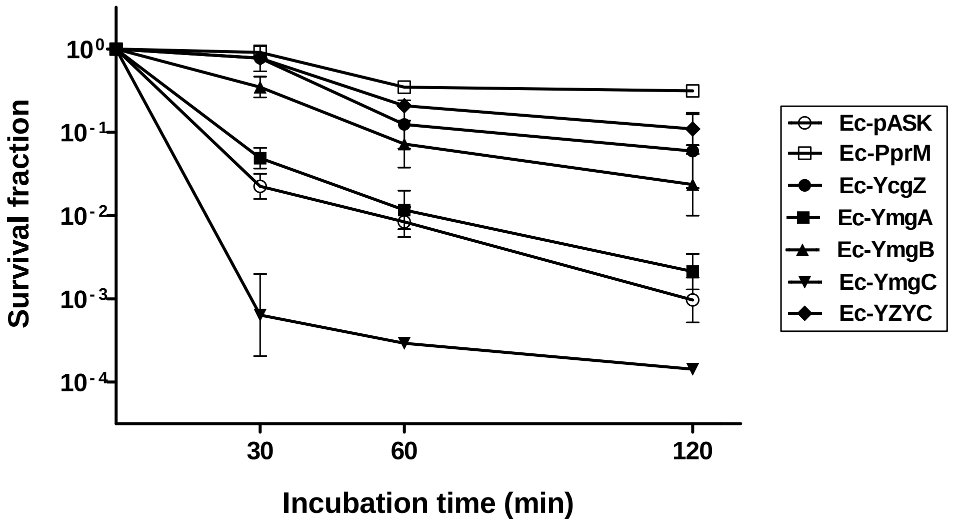

Figure S3. Survival of *E. coli* expressing the *ycgZ*, *ymg*, and *pprM* genes under acidic conditions. Following the 2 h incubation with AHT (200 ng/ml), the recombinant strains were incubated in LB medium (pH 2.5) for indicated times. The survival fraction was calculated by dividing the CFUs of acid-treated cells by the CFUs of non-treated cells. The error bars represent the standard deviation of three independent experiments (n=3).
